# Sentinel node biopsy in early vulvar cancer

**DOI:** 10.1054/bjoc.1999.0918

**Published:** 2000-01-18

**Authors:** C De Cicco, M Sideri, M Bartolomei, C Grana, M Cremonesi, M Fiorenza, A Maggioni, L Bocciolone, C Mangioni, N Colombo, G Paganelli

**Affiliations:** Division of Gynaecology and Division of Nuclear Medicine, European Institute of Oncology, Via G. Ripamonti, Milan, 435-I-20141, Italy; Division of Gynaecology, S. Gerardo Hospital, Monza, Italy

**Keywords:** vulvar cancer, lymph node, lymphoscintigraphy, sentinel node, lymphadenectomy

## Abstract

Lymph node pathologic status is the most important prognostic factor in vulvar cancer; however, complete inguinofemoral node dissection is associated with significant morbidity. Lymphoscintigraphy associated with gamma-probe guided surgery reliably detects sentinel nodes in melanoma and breast cancer patients. This study evaluates the feasibility of the surgical identification of sentinel groin nodes using lymphoscintigraphy and a gamma-detecting probe in patients with early vulvar cancer. Technetium-99m-labelled colloid human albumin was administered perilesionally in 37 patients with invasive epidermoid vulvar cancer (T1–T2) and lymphoscintigraphy performed the day before surgery. An intraoperative gamma-detecting probe was used to identify sentinel nodes during surgery. A complete inguinofemoral node dissection was then performed. Sentinel nodes were submitted separately to pathologic evaluation. A total of 55 groins were dissected in 37 patients. Localization of the SN was successful in all cases. Eight cases had positive nodes: in all the sentinel node as positive; the sentinel node was the only positive node in five cases. Twenty-nine patients showed negative sentinel nodes: all of them were negative for lymph node metastases. Lymphoscintigraphy and sentinel-node biopsy under gamma-detecting probe guidance proved to be an easy and reliable method for the detection of sentinel node in early vulvar cancer. This technique may represent a true advance in the direction of less aggressive treatments in patients with vulvar cancer. © 2000 Cancer Research Campaign


*British Journal of Cancer* (2000) **82**(2), 295–299
© 2000 Cancer Research Campaign
Article no. bjoc.1999.0918

### Sentinel node biopsy in early vulvar cancer

**C De Cicco[2], M Sideri[1], M Bartolomei[2], C Grana[2], M Cremonesi[2], M Fiorenza[2], A Maggioni[1], L Bocciolone[1], C Mangioni[3], N Colombo[1] and G Paganelli[2]**

[1]Division of Gynaecology and [2]Division of Nuclear Medicine, European Institute of Oncology, Via G. Ripamonti, 435-I-20141 Milan, Italy; [3]Division of Gynaecology, S. Gerardo Hospital, Monza, Italy

**Summary** Lymph node pathologic status is the most important prognostic factor in vulvar cancer; however, complete inguinofemoral node dissection is associated with significant morbidity. Lymphoscintigraphy associated with gamma-probe guided surgery reliably detects sentinel nodes in melanoma and breast cancer patients. This study evaluates the feasibility of the surgical identification of sentinel groin nodes using lymphoscintigraphy and a gamma-detecting probe in patients with early vulvar cancer. Technetium-99m-labelled colloid human albumin was administered perilesionally in 37 patients with invasive epidermoid vulvar cancer (T1–T2) and lymphoscintigraphy performed the day before surgery. An intraoperative gamma-detecting probe was used to identify sentinel nodes during surgery. A complete inguinofemoral node dissection was then performed. Sentinel nodes were submitted separately to pathologic evaluation. A total of 55 groins were dissected in 37 patients. Localization of the SN was successful in all cases. Eight cases had positive nodes: in all the sentinel node was positive; the sentinel node was the only positive node in five cases. Twenty-nine patients showed negative sentinel nodes: all of them were negative for lymph node metastases. Lymphoscintigraphy and sentinel-node biopsy under gamma-detecting probe guidance proved to be an easy and reliable method for the detection of sentinel node in early vulvar cancer. This technique may represent a true advance in the direction of less aggressive treatments in patients with vulvar cancer. © 2000 Cancer Research Campaign

**Key words**: vulvar cancer; lymph node; lymphoscintigraphy; sentinel node; lymphadenectomy

Vulvar cancer accounts for 0.5% of all malignancies; the most frequent histologic type is squamous cell carcinoma, which affects predominantly elderly women. From 1940 radical vulvectomy with bilateral inguinofemoral lymphadenectomy has been the standard treatment for these patients. This operation entails complete removal of the vulva, mons pubis, inguinofemoral nodes and sometimes pelvic nodes. Lymphadenectomy may result in high morbidity, with wound breakdown, wound infection occurring in up to 85% of the cases and chronic lymphoedema reported in 15–20% of the patients (Podratz et al, 1982).

In the last 10 years the surgical procedure has become more conservative (Sutton et al, 1991; Cavanagh, 1997) and in patients with early cancer a modified radical vulvectomy or a wide excision of the tumour was shown to be adequate to eradicate the local vulvar lesion. However, although groin dissection is considered necessary in patients with clinically positive nodes, the procedure may be an overtreatment when inguinal nodes are free of disease. A reduction of the surgical aggressiveness in the groin has been attempted, resulting sometimes in an increase of recurrence rate (Stehman et al, 1992; Burke et al, 1995). In 1977, Cabanas introduced the sentinel node (SN) concept for penile carcinoma, suggesting to select subjects for complete lymphadenectomy (Cabanas, 1977). In 1979, Di Saia et al, considering the first level of inguinofemoral lymph nodes, superficial nodes, as the first draining site in vulvar cancer, proposed a limited dissection of these nodes if they resulted free of disease (Di Saia et al, 1979). However, a Gynaecology Oncology Group (GOG) controlled clinical trial demonstrated a decrease of disease-free survival and increase in recurrence rate when this technique was applied in a large multicentric study (Stehman et al, 1992).

In 1992, Morton et al applied the concept of SN in cutaneous melanoma (Morton et al, 1992*a*, 1992*b*), employing the blue dye technique in order to identify SN for biopsy. Levenback et al (1994, 1995) applied this technique in vulvar squamous cell cancer, finding that SN status was predictive of the whole groin node pathologic status. However, with blue dye the SN was missed in 34% of the evaluated groins and no lymphatic channel was identified in 24% of the patients.

Lymphoscintigraphy is another method for localizing SN; it is a non-invasive and well-established technique for evaluating the anatomical pattern of lymphatic distribution and it has been proven to be a suitable method to study the lymphatic drainage pathways of the vulva (Iversen and Aas, 1983, Barton et al, 1992). Associated with gamma-probe guided surgery, lymphoscintigraphy has recently been proposed as a more suitable technique than blue dye injection to remove during surgery the sentinel nodes in melanoma and breast cancer patients (Alex et al, 1993; Krag et al, 1993). In these patients the reliability and accuracy of the procedure has been demonstrated by several studies (Van der Veen et al, 1994; Veronesi et al, 1997), while some preliminary reports on ten patients showed that lymphoscintigraphic method is feasible for the identification of the SN in squamous cell cancer of the vulva as well (De Cesare et al, 1997; De Hullu et al, 1998).

The present study is proposed in order to verify the predictive value of the SN status in a larger series of patients.

*Received 27 April 1999*
*Revised 8 September 1999*
*Accepted 14 September 1999*

*Correspondence to:* C De Cicco

**Table 1** Summary of sentinel node findings in 37 vulvar lesions

| Primary lesion site | No. pts | SN detection (LS plus GPD) | Groin(s) involved | | No. of lymph nodes studied | |
|---|---|---|---|---|---|---|
| | | | Ipsilat. | Bilat. | SN | non-SN |
| Lateral | 19 | 100% | 19 | – | 30 (6*) | 219 (6*) |
| Midline | 18 | | 5 | 13 | 49 (4*) | 408 (1*) |
| Total | 37 | | 50 | | 79 (10*) | 627 (7*) |

(*) = No. of positive lymph nodes.

### METHODS

### Patients and administration technique

From May 1996 to September 1998, 37 consecutive patients with early invasive squamous-cell vulvar cancer were studied. Patients with clinically positive groin nodes, pregnant or lactating patients were excluded from the study. Thirty-three patients presented a primary tumour; four patients had a previous diagnostic surgical excision of the lesion. Seventeen patients had T1 and 20 had T2 vulvar lesions, without clinical evidence of regional lymph-node metastases. Nineteen patients had unilateral lesions and 18 patients had midline lesions (Table 1). A midline lesion is defined as a tumour located within 2 cm of the midline. An informed consent for the participation in the study was obtained from all the patients.

Lymphoscintigraphy was performed the day before surgery to establish lymphatic drainage and to define the location of the sentinel nodes. Colloid particles of human albumin ranging from 50 to 80 nm in size (Nanocoll, supplied by Amersham-Sorin, Saluggia, Italy) were used. The colloids were labelled with $^{99m}$Tc freshly eluted from a generator and submitted to a quality control for free technetium according with manufacturer recommendations. Radiochemical purity of the tracers was always over 95%. Generally 5 MBq of $^{99m}$Tc-colloids per injection in a volume of 0.1 ml were administered subcutaneously at the junction of the tumour and the normal skin, through a 25-gauge needle, followed by 0.1 ml of air in order to ensure the complete tracer administration. The number of injections ranged from two to four, depending on the site and shape of the lesions. The total dose administered to each patient was 10–20 MBq. When the lesion was clearly unilateral the administration of the tracer was performed in two sites close to the tumour (at 6 and 12 o'clock positions); in cases of lesions near to midline four injections around the tumour at 3, 6, 9, 12 o'clock positions were done.

### Scintigraphic studies

Planar scans of the vulvar and inguinal areas in anterior and lateral projections were obtained after 5–20 min (early) and 3 h (late) from radiotracer injection (Figures 1 and 2). Each acquisition lasted 5 min. Delayed images were acquired when no drainage was documented. After acquisition of the last scan, a cutaneous marker with a suitable pen was positioned in correspondence to the first lymph node chronologically revealed by the gamma-camera.

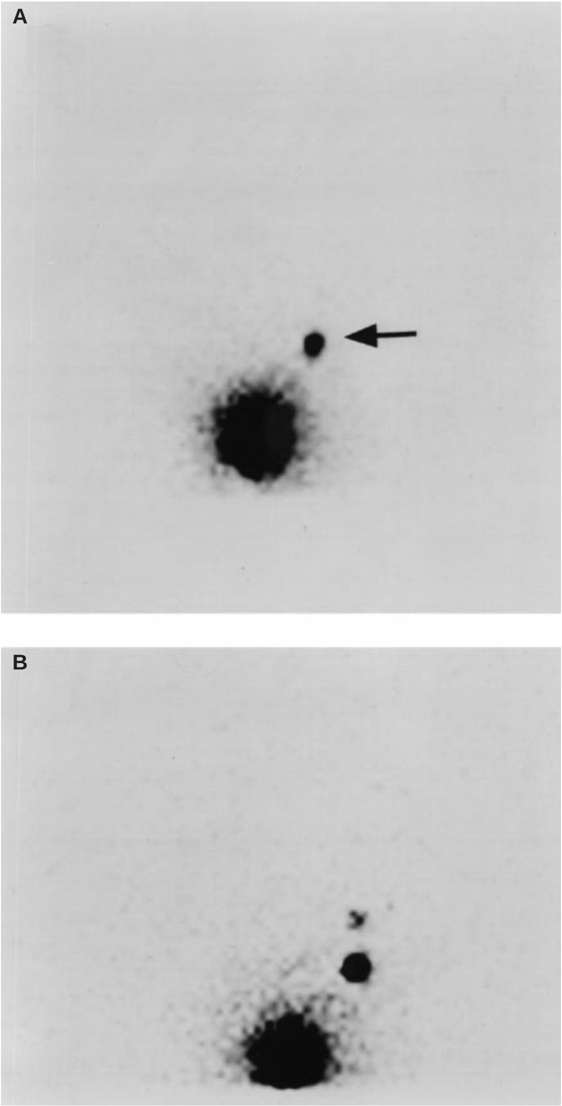

**Figure 1** Lymphoscintigraphy in patient with a midline vulvar lesion reveals an intensively radioactive sentinel node (arrow) in the left groin in the early image (**A**). A second less intense radioactive focus is cleared 3 h post injection (**B**). No sentinel nodes are revealed in the right side. The injection site is on the lower of the figure

 *© 2000 Cancer Research Campaign*

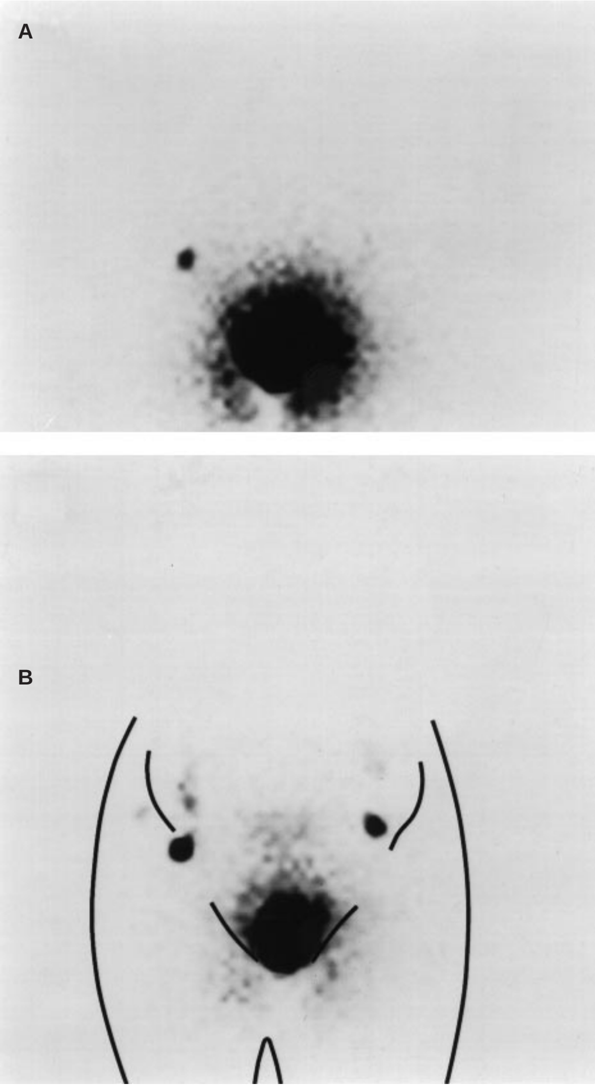

**Figure 2** Early (**A**) and delayed (**B**) scintigraphic images of the pelvis in anterior view in patient with a tumoural lesion of the midline. In A only a sentinel node in the right side is revealed; sentinel node of the controlateral groin and some (several) bilateral faint foci of uptake are evident in **B**

### Surgical procedure

A standard surgical approach, consisting of wide radical excision of the tumour, hemivulvectomy or radical vulvectomy, was used in all patients. For unilateral lesions equal to or less than 2 cm in diameter ipsilateral groin dissection was performed. When the lesion was greater than 2 cm or in the midline, bilateral groin dissection was done.

Before groin dissection was carried out, the gamma-detecting probe (GDP) MR 100, from Po.li.tech. (L'Aquila, Italy), was inserted in a sterile glove and used by the surgeon to localize the area emitting the higher signal of gamma-ray emission; then a 3 cm incision was made and SN excised and labelled as 'sentinel' lymph node. When more than one SN was detected, the nodes were ordered on the basis of radioactivity levels and sent separately to the pathologist. A complete groin dissection was then performed, according to Micheletti technique (Micheletti et al, 1990): superficial lymph nodes contained in the fat pad along the inguinal ligament and deep femoral nodes lying below and above the junction of the saphenous and femoral veins, with preservation of the cribriform fascia, were removed. Then the GDP was slowly passed over the removed surgical specimen, in order to exclude the presence of other gamma-emitting foci in it.

### Pathologic examination

A pathologist examined all the surgically-removed groin specimens using standard technique. Briefly, the lymph nodes were isolated fresh from groin fat tissue. Nodes >0.5 cm in diameter were divided in two parts along the longitudinal axis, whereas nodes <0.5 cm in the major axis were embedded in toto. Three histological sections were obtained from each inclusion, at different cut levels (at a distance between 0.3 and 1 mm), and were stained with haematoxylin and eosin.

### RESULTS

In all cases lymphoscintigraphy detected at least one lymph node within 3 h after tracer administration. The mean number of nodes localized was 1.7 in unilateral lesions; in 18 patients with midline lesion an average of 3.3 SNs were detected (1.7 per groin). In 13 out of the 18 patients with midline lesions the SN was identified in

**Table 2** Pathologic results in the group of 8 patients with groin involvement

| | | Eight out 37 patients with histologically positive SN | | | | | | | |
|---|---|---|---|---|---|---|---|---|---|
| | | 1 | 2 | 3 | 4 | 5 | 6 | 7 | 8 |
| Site of the tumour (R, M, L) | | R | L | L | R | M | M | M | M |
| Size of the tumour (Ø mm) | | 20 | 30 | 8 | 35 | 60 | 20 | 3 | 12 |
| Depth invasion (mm) | | 15 | 4 | 7 | 10 | 13 | 23 | 3 | 9 |
| FIGO stage | | III | III | III | III | III | III | III | III |
| No. of SN(s) | | 1 | – | – | 2 | 1 | 1 | 0 | 1 |
| | Right groin | (1*) | | | (2*) | (0*) | (1*) | | (0*) |
| | | – | 1 | 2 | – | 2 | 3 | 1 | 2 |
| | Left groin | | (1*) | (2*) | | (1*) | (0*) | (1*) | (1*) |
| No. of non-SNs | | 9 | – | – | 8 | 6 | 8 | 9 | 9 |
| | Right groin | (0*) | | | (1*) | (0*) | (0*) | (0*) | (0*) |
| | | – | 13 | 13 | – | 10 | 20 | 7 | 8 |
| | Left groin | | (0*) | (5*) | | (1*) | (0*) | (0*) | (0*) |

R = right; M = midline; L = left. (*) = No. of positive lymph nodes.

*© 2000 Cancer Research Campaign*

both groins. In five patients no contralateral lymphatic drainage was documented. In most cases (33/37, 89%) the SN was visualized in less than 30 min and in four cases in the late images; in two of these patients delayed images were required (later than 10 h post-injection). The mean number of SN surgically detected with the GDP per groin was 1.4 (range 0–4). The total number of nodes removed was 736 (mean 13; range 9–23) (Table 1).

Eight out of 37 cases had positive nodes (Table 2); the SN was the only positive node in six cases; in the remainder three, in one case two SNs and one non-SN were positive; in one patient the SN was one of two positive nodes, while in the last case two SNs and other five non-SN were found to have metastases. Twenty-nine patients showed negative SN: all of them were histologically negatives. In the five out of 18 patients with midline lesion, with only one groin showed lymphatic drainage, controlateral lymphadenectomy did not show metastatic nodes.

### Dosimetry

The absorbed dose to the inoculated area and lymph nodes were estimated at 0.85 ± 0.5 mGy $MBq^{-1}$ and 0.05 ± 0.03 mGy $MBq^{-1}$ respectively. Because of the small quantity of material injected and the fact that it was mainly concentrated in tissue that was removed, the adsorbed dose to the site of injection and other tissue was negligible. The air kerma rate at 0, at 50 and at 100 cm from the injection site immediately before surgery (about 20 h after injection) was 4.5 ± 1.0, 1.5 ± 0.4 and 0.9 ± 0.3 μGy $h^{-1}$ respectively. The absorbed dose to the surgeons' hands after 100 cases using 20 MBq per case would be 9.0 ± 1.6 μSv, corresponding to less than 2% and 0.2% of the recommended limits for the population and the exposed personnel respectively (ICPR, 1991).

The procedure is therefore safe and does not pose any radiation risk for patients, hospital staff, relatives or surgical team.

### DISCUSSION

Sentinel-node biopsy by means of GDP after lymphoscintigraphy proved to be an accurate technique in melanoma and breast cancer patients, in order to predict lymph node status. Several studies performed using both blue dye and lymphoscintigraphic methods demonstrated so far that the sentinel node concept is functionally valid and may avoid in the near future many lymphadenectomies in early cancer (Albertini et al, 1996; Veronesi et al, 1997; Bartolomei et al, 1998; Krag et al, 1998). Sentinel node concept seems to be applicable to vulvar cancer as well: Levenback in two consecutive series of 9 and 21 patients studied with blue dye technique found that in no case was there a regional node positive when the sentinel node was negative. However, the blue dye method is affected by a low sensitivity in identifying the sentinel node. Recently, two different preliminary studies in vulvar cancer (De Cesare et al, 1997; De Hullu et al, 1998), both with ten patients examined, had optimal results applying lymphoscintigraphy intraoperatively and the day before surgery respectively. In both of them sensitivity and predictive value of the procedure were 100%. In the same way in our series of 37 patients with early squamous cancer the status of the sentinel node was predictive of the groin status in all cases and in five out of eight cases in which inguinal nodes were involved, the sentinel node was the only metastatic node. Furthermore, in two out of three cases in which more than one node was found to be metastatic both of two SNs were positive for metastases.

In 18 patients with midline lesions we never found positive nodes in the groin in which no sentinel nodes were identified (five patients). This finding indicates that in these conditions it is safe to avoid every surgical procedure in the groin in which no lymphatic drainage is documented at scan. In regard to the method, we recommend performing lymphoscintigraphy the day before surgery, in order to avoid an uncertain outcome about sentinel node localization and the presence of drainage in each side of the inguinal region as well. Nevertheless in 90% of the patients sentinel nodes are visualized in less than 15 min and in fact tracer administration could be done immediately before surgery. Indeed, De Cesare et al (1997) used intraoperative administration of the radiotracer with high sensitivity. However, in our experience lymphatic drainage could occur later after administration, rendering the procedure useless, particularly in patients who were previously treated with partial surgical excision of the primary lesion or with laser therapy. However, in our series including four patients in this condition, surgical and laser treatments have not affected the procedure and no misleading results were found in patients previously treated.

We identified some potential limitations to the method:

- Although in our series no patients refused the injection of the tracer, painful lesions could make the injection procedure less tolerable; anaesthetic cream application 1 h before administration can resolve this problem.
- The high vascularity of the region and lesions can often produce bleeding and blood vessel injection; this produces a high background rate, which can disturb identification of the nodes by the probe during surgery. It is important to avoid intratumoural administration, injecting the tracer superficially around the tumour and performing aspiration after puncture.

In summary, lymphoscintigraphy seems to be a feasible and well-tolerated procedure for sentinel node localization, although skilled operators are required. The technique is simple and is ideally suited to be used in large multicentre studies to test the predictivity of sentinel-node biopsy in early vulvar cancer. Combining previous published data (Levenback et al, 1995; De Cesare et al, 1997; De Hullu et al, 1998) with the present ones, SN status was predictive of groin node positivity in a total of 78 patients. This result encourages the performance of a study on selective lymphadenectomy in these patients. If we are successful in staging groin lymph nodes by a simple biopsy, we could eliminate the risk of complications associated with a complete inguinofemoral node dissection for most patients and achieve considerable savings in costs and morbidity.

In conclusion, sentinel-node biopsy under GDP guidance proved to be an accurate method for intraoperative detection of sentinel nodes. This technique may represent a true advance in the direction of less aggressive treatments in patients with vulvar cancer.

### REFERENCES

Albertini JJ, Lyman GH, Cox C, Yeatman T, Balducci L, Ku NN, Shivers S, Berman C, Wells K, Rapaport D, Shons A, Horton J, Greenberg H, Nicosia S, Clark R, Cantor A and Reitgen DS (1996) Lymphatic mapping and sentinel node biopsy in the patient with breast cancer. *JAMA* **276**: 1818–1822

Alex JC, Weaver DL and Fairbank JT (1993) Gamma-probe-guided lymph node localisation in malignant melanoma. *Surg Oncol* **2**: 303–308

© 2000 Cancer Research Campaign

Bartolomei M, Testori A, Chinol M, Gennari R, De Cicco C, Leonardi L, Zoboli S and Paganelli G (1998) Sentinel node localization in cutaneous melanoma: lymphoscintigraphy with colloids and antibody fragments versus blue dye mapping. *Eur J Med* **25**: 1489–1494

Barton DPJ, Berman C, Cavanagh D, Roberts WS, Hoffman MS, Fiorica and Finan MA (1992) Lymphoscintigraphy in vulvar cancer: a pilot study. *Gynecol Oncol* **46**: 341–344

Burke TW, Levenback C, Coleman RL, Morris M, Silva EG and Gershenson DM (1995) Surgical therapy of T1 and T2 vulvar carcinoma: further experience with radical wide excision and selective inguinal lymphadenectomy. *Gynecol Oncol* **57**: 215–220

Cabanas RM (1977) An approach for the treatment of penile carcinoma. *Cancer* **39**: 456–466

Cavanagh D (1997) Vulvar cancer: continuing evolution in management. *Gynecol Oncol* **66**: 362–367

De Cesare SL, Fiorica JV, Roberts WS, Reintgen D, Arango H, Hoffman MS, Puleo C and Cavanagh D (1997) A pilot study utilizing intraoperative lymphoscintigraphy for identification of the sentinel nodes in vulvar cancer. *Gynecol Oncol* **66**: 425–428

De Hullu JA, Doting E, Piers DA, Hollem H, Aalders JG, Schraffords Koops H, Boonstra H and van der Zee AGJ (1998) Sentinel lymph node identification with technetium-99m-labeled nanocolloid in squamous cell cancer of the vulva. *J Nucl Med* **39**: 1381–1385

Di Saia PJ, Creasman WT and Rich WM (1979) An alternate approach to early cancer of the vulva. *Am J Obstet Gynecol* **133**: 825–830

ICRP (1991) International Commission on Radiological Protection, Publication 60. Pergamon Press: Oxford

Iversen T and Aas M (1983) Lymph drainage from the vulva. *Gynecol Oncol* **16**: 179–189

Krag D, Weaver D, Ashikaga T, Moffat F, Klinberg VS, Shriver C, Feldman S, Kusminky R, Gadd M, Kuhn J, Harlow S and Beitsch P (1998) The sentinel node in breast cancer. A multicenter validation study. *N Engl J Med* **339**: 941–946

Levenback C, Burke TW, Gershenson DM, Morros M, Malpica A and Ross MI (1994) Intraoperative lymphatic mapping for vulvar cancer. *Obstet Gynecol* **84**: 163

Levenback C, Burke TW, Morris M, Malpica A, Lucas KR and Gershenson DM (1995) Potential applications of intraoperative lymphatic mapping in vulvar cancer. *Gynecol Oncol* **59**: 216

Micheletti L, Borgno G, Barbero M, Preti M, Nicolaci P, Benedetto C, Ghiringhello B and Bocci A (1990) Deep femoral lymphadenectomy with preservation of the fascia lata. Preliminary report on 42 invasive vulvar carcinomas. *J Reprod Med* **35**: 1130–1133

Morton D, Wen D and Cochran A (1992*a*) Management of early-stage melanoma by intraoperative lymphatic mapping and selective lymphadenectomy: an alternative to routine elective lymphadenectomy or ‘watch and wait’. *Surg Oncol Clin North Am* **1**: 247–259

Morton D, Wen D, Wong JH, Econoumou JS, Cagle LA, Storm FK, Foshag LJ and Cochran AJ (1992*b*) Technical details of intraoperative lymphatic mapping for early stage melanoma. *Arch Surg* **127**: 392–399

Podratz KC, Symmonds RE and Taylor F (1982) Carcinoma of the vulva: analysis of treatment failures. *Am J Obstet Gynecol* **143**: 340–351

Stehman FB, Bundy BN, Dvoretsky PM and Creasman WT (1992) Early stage I carcinoma of the vulva treated with ipsilateral superficial inguinal lymphadenectomy and modified radical hemivulvectomy: a prospective study of the Gynaecologic Oncology Group. *Obstet Gynecol* **79**: 490

Sutton GP, Miser MR, Stehman FB, Look KY and Ehrlich CE (1991) Trends in operative management of invasive squamous carcinoma of the vulva at Indiana University, 1974 to 1988. *Am J Obstet Gynecol* **164**: 1472

Van der Veen H, Hoekstra OS, Paul MA, Cuesta MA and Meijer S (1994) Gamma probe-guided sentinel node biopsy to select patients with melanoma for lymphadenectomy. *Br J Surg* **81**: 1769–1770

Veronesi U, Paganelli G, Galimberti V, Viale G, De Cicco C, Luini A, Sacchini V, Veronesi P and Zurrida S (1997) Sentinel node biopsy can avoid axillary dissection in breast cancer patients with clinically negative lymph nodes. *Lancet* **349**: 1864–1867

*© 2000 Cancer Research Campaign*
